# Are Pathogenic Germline Variants in Metastatic Melanoma Associated with Resistance to Combined Immunotherapy?

**DOI:** 10.3390/cancers12051101

**Published:** 2020-04-28

**Authors:** Teresa Amaral, Martin Schulze, Tobias Sinnberg, Maike Nieser, Peter Martus, Florian Battke, Claus Garbe, Saskia Biskup, Andrea Forschner

**Affiliations:** 1 Center for Dermatooncology, Department of Dermatology, University Hospital Tuebingen, Eberhard Karls University, 72076 Tuebingen, Germany; Teresa.amaral@med.uni-tuebingen.de (T.A.); Tobias.Sinnberg@med.uni-tuebingen.de (T.S.); Claus.Garbe@med.uni-tuebingen.de (C.G.); 2 Portuguese Air Force, Health Care Direction, 1649-020 Lisbon, Portugal; 3 Practice for Human Genetics, 72076 Tuebingen, Germany; martin.schulze@humangenetik-tuebingen.de (M.S.); Maike.Nieser@humangenetik-tuebingen.de (M.N.); Saskia.Biskup@humangenetik-tuebingen.de (S.B.); 4 Institute for Clinical Epidemiology and applied Biostatistics (IKEaB), Eberhard Karls University, 72076 Tuebingen, Germany; Peter.Martus@med.uni-tuebingen.de; 5 Center for Genomics and Transcriptomics (CeGaT) GmbH, 72076 Tuebingen, Germany; Florian.Battke@cegat.de

**Keywords:** pathogenic/likely pathogenic germline variant, immune checkpoint inhibitors, advanced melanoma, resistance to immunotherapy

## Abstract

Background: Combined immunotherapy has significantly improved survival of patients with advanced melanoma, but there are still patients that do not benefit from it. Early biomarkers that indicate potential resistance would be highly relevant for these patients. Methods: We comprehensively analyzed tumor and blood samples from patients with advanced melanoma, treated with combined immunotherapy and performed descriptive and survival analysis. Results: Fifty-nine patients with a median follow-up of 13 months (inter quartile range (IQR) 11–15) were included. Interestingly, nine patients were found to have pathogenic or likely pathogenic (P/LP) germline variants in one of these genes: BRCA2, POLE, WRN, FANCI, CDKN2A, BAP1, PALB2 and RAD54B. Most of them are involved in DNA repair mechanisms. Patients with P/LP germline variants had a significantly shorter progression-free survival (PFS) and melanoma specific survival (MSS) compared to patients without P/LP germline variants (HR = 2.16; 95% CI: 1.01–4.64; *p* = 0.048 and HR = 3.21; 95% CI: 1.31–7.87; *p* = 0.011, respectively). None of the patients with a P/LP germline variant responded to combined immunotherapy. In the multivariate Cox-regression analysis, presence of a P/LP germline variant, S100B and lactate dehydrogenase (LDH) remained independently significant factors for MSS (*p* = 0.036; *p* = 0.044 and *p* = 0.001, respectively). Conclusions: The presence of P/LP germline variants was associated with resistance to combined immunotherapy in our cohort. As genes involved in DNA repair mechanisms are also involved in lymphocyte development and T-cell differentiation, a P/LP germline variant in these genes may preclude an antitumor immune response.

## 1. Introduction

Cancer is a genetic disease and in the last decades, a huge effort has been made to uncover the implications of genetics in cancer [1,2]. With the increasing availability of next-generation sequencing (NGS) of tumor tissue of cancer patients, a growing body of knowledge has become available. This includes information on somatic variants and the relevance of their total number expressed as tumor mutational burden (TMB) [3,4], and also on pathogenic germline variants identified in different cancer landscapes beyond the well-studied familial cancer syndromes [5,6,7,8,9]. Current methods of tumor DNA sequencing allow the identification of potentially targetable alterations that might have implications in designing new therapies that improve patients’ outcomes. In the last years, anti-PD-1 based immunotherapy has become a part of the standard of care in the advanced setting for cancer patients, particularly for cutaneous melanoma [10,11,12,13]. Additionally, we have experienced a clear shift from nonspecific chemotherapy to a more personalized approach, based not only on the tumor genetic alterations [14,15,16], for example BRAF/MEK inhibition in melanoma patients [17,18], poly-(ADP) ribose polymerase inhibitors (PARPi) in patients with pathogenic germline *BRCA1/2* variants [19,20], but also on the individual patients’ characteristics [21,22,23].

Pathogenic germline variants have been found commonly in a variety of tumors from patients that have undergone tumor and normal tissue sequencing [9,24]. In this work, we identified pathogenic and likely pathogenic (P/LP) germline variants in a cohort of patients with advanced melanoma (stage IV of the American Joint Committee on Cancer (AJCC) 8th Edition [25]) and treated with combined immunotherapy (nivolumab and ipilimumab). Germline variants were classified according to the American College of Medical Genetics and Genomics (ACMG) standards and guidelines for the interpretation of sequence variants, representing the gold standard classification system widely used in clinical genetic diagnostics. Here, we focus on high impact germline variants assigned pathogenic or likely pathogenic according to ACMG guidelines [26] and their potential impact on therapy outcome. For RAD54B, a gene involved in homologous recombination the OMIM database (OMIM *604289) currently only lists somatic variants to be of relevance in cancer. However, Zhao et al. [27] described pathogenic germline mutations in RAD54B to be of potentially disease relevance in a Chinese cohort of ovarian cancer patients. Based on this finding, and due the role of RAD54B in homologous repair, we consider RAD54B to represent an important candidate gene in which P/LP germline variants are likely of familial and therapeutic relevance, even though the ACMG criteria is not formally intended to be used to classify variants in genes without (an established/a known) hereditary phenotype.

With the previous considerations, we went on investigating whether the presence of these P/LP germline variants are associated with survival and response to systemic therapy, particularly to combined immunotherapy (nivolumab plus ipilimumab).

## 2. Materials and Methods

### 2.1. Patients

In the current analysis, we included all 59 patients who had been enrolled in a prospective study on the value of liquid biopsy and next-generation sequencing and who received combined immunotherapy in the period following enrollment. The patients had a diagnosis of stage IV melanoma, and clinical indication for treatment with systemic therapy. Patients were included only if tumor and normal tissue were available for sequencing. Written consent for study participation was obtained from all patients. Informed consent was also given according to the Gene Diagnostic Law in Germany. The sequencing results were reported to the patients and assisting physician, according to their preferences. Ethical approval was obtained from both the Aerztekammer Baden-Wuerttemberg and the local ethics committee of the Eberhard Karls University (approval numbers F-2016-010 and 827/2018BO2). This study was performed in accordance with the Declaration of Helsinki.

### 2.2. DNA Extraction, Sequencing and Computational Analysis

For all somatic analyses, DNA from blood was sequenced in parallel as the corresponding normal tissue control. Formalin-fixed paraffin-embedded (FFPE) blocks from the most recently excised metastatic tissue were used for sequencing. Germline mutations were always determined from both tumor and normal tissue. DNA was isolated from FFPE material using black PREP FFPE DNA Kit (Analytik Jena, Jena, Germany). The coding region and flanking intronic regions of 710 tumor relevant genes (CeGaT inhouse design, Supplementary Materials supplement 1) were enriched using in solution hybridization technology (Agilent, Santa Clara, CA, USA or TWIST Bioscience, San Francisco, CA, USA) and were sequenced using the Illumina HiSeq/NovaSeq system (Illumina, San Diego, CA, USA) with an average coverage of 575 reads per base (SE 234.4). Illumina bcl2fastq2 (Version 2.20.0.422, Illumina Inc.) was used to demultiplex sequencing reads. Adapter removal was performed with Skewer (Skewer 0.2.2) [28]. The trimmed reads were mapped to the human reference genome (hg19) using the Burrows Wheeler Aligner (bwa 0.7.2-r351) [29]. Reads mapping to more than one location with identical mapping score were discarded. Read duplicates that likely result from PCR amplification and reads mapping to more than one genomic location were removed. The remaining high-quality sequences were used to determine sequence variants (single nucleotide changes and small insertions/deletions). Only variants (single nucleotide variants (SNVs)/small indels) in the coding region and the flanking intronic regions (±8 bp) with a minor allele frequency (MAF) < 1.5% were evaluated. Known disease-causing variants (according to The Human Gene Mutation Database HGMD® [30]) were evaluated in up to ±30 bp of flanking regions and up to 5% MAF. Minor allele frequencies were taken from public databases (gnomAD and dbSNP) and an in-house database. Copy number variations (CNV) were computed on uniquely mapping, non-duplicate, high quality reads using an internally developed method based on sequencing coverage depth. Briefly, we used reference samples to create a model of the expected coverage that represents wet-lab biases as well as intersample variation. CNV calling was performed by computing the sample’s normalized coverage profile and its deviation from the expected coverage. Genomic regions were called as variant if they deviate significantly from the expected coverage. The expected coverage for each exon is computed from the reference population as the median of the exon-level coverages of each reference sample as follows: Expected coverage for one sample is computed as a number of reads mapping to each exonic region, normalized by total number of on-target reads (as reads-per-million) to remove influences by different enrichment efficiencies. Read counts are further normalized by the expected number of alleles present (2 for all autosomes, 0/1/2 for gonosomes as determined by patient sex), resulting in read-per-million-per-allele (RPMA) values. For each exon, the median RPMA value in the reference population as well as the MAD (median average deviation) is computed. Exon-level RPMA values are also computed for the sample of interest. These are compared to the reference median RPMA resulting in a percentage deviation (e.g., twice as much as expected, half as much as expected) as well as a z-score (as (observed RPMA-expected median RPMA)/expected MAD RPMA). Exons are called as variant if they deviate by at least 2 standard deviations from the model mean (i.e., z-score is ≤−2 or ≥2) and the deviation is concordant with a biologically possible copy number (e.g., +50% for a heterozygous duplication, −50% for a heterozygous deletion).

#### 2.2.1. Tumor Mutation Burden

The methods used for determination of the tumor mutational burden based on comparative sequencing of DNA from tumor and normal tissue were already described elsewhere [31]. TMB was defined as the number of somatic single nucleotide variants, InDel and essential splice changes in the entire coding region (exome) and as mutations (Mut) per million coding bases (Mb). To calculate the tumor mutation burden, firstly somatic variants were counted which affect the protein coding regions of all sequenced genes (synonymous as well as non-synonymous) with a minimal variant frequency of 10%. The variants identified by sequencing 710 gene panels were divided into driver and passenger mutations, and the resulting two counts were used to estimate the number of somatic variants in the entire exome. For this estimation, it was assumed that the passenger mutations occur in all known genes at the same density, i.e., their number was upscaled relative to the difference between the size of the gene panel and the size of the whole exome. It was assumed that the driver mutations are restricted to tumor-associated genes, and their number was not upscaled. The estimated total number of passenger and driver mutations was normalized to the size of the total coding exome. The classification of TMB is usually made in the following categories: "low" (<3.3 mut/Mb) “intermediate” (3.3–23.1 mut/Mb) and "high” (>23.1 mut/Mb) [32,33]. For this study patients were dichotomized into the two sub-groups: intermediate-low TMB (≤23.1 mut/Mb) and high TMB (>23.1 mut/Mb).

#### 2.2.2. Clinical and Biological Interpretation of Germline Alterations, Classification and Assessment of Potential Therapies

For known disease-associated genes, variant classification was performed according to ACMG guidelines [26]. For this study, class 5 (pathogenic), class 4 (likely pathogenic) and variants in genes, which represent risk factors, or which can be of relevance for treatment decision, were reported as P/LP germline variants (Table 1). For each P/LP germline variant, a possible “off-label” treatment option is given in Table 1, considering known interferences and pathways [34,35,36,37] as well as level of evidence. The underlying classification in view of the level of evidence (LOE) reported in Table 1 can be found in detail in Supplementary Materials supplement 2. Please note, the LOE classification reported here is adapted from the LOE classification established by the oncology knowledge base (OncoKB) [38]. For biological interpretation, the corresponding protein names of the nine affected genes were loaded into the STRING Protein–Protein Interaction Networks Functional Enrichment Analysis tool using the mask ‘multiple proteins’ [39]. All reported gene ontology (GO) annotated biological processes have a false discovery rate <0.0005.

### 2.3. Statistical Methods

Only patients for whom a P/LP germline variant was identified were included in the group of patients with germline variants. Patients with only somatic variants were included in the “non-germline variant group”. Categorical data were compared using the exact version of the Chi-square test as implemented in the statistical software SPSS v.25 (SPSS Inc., Chicago, IL, USA).

The follow-up (FU) time was defined as the time between the beginning of combined immunotherapy and date of patients’ last contact or death from any cause. For the calculation of melanoma specific survival (MSS) only deaths due to melanoma were considered events. MSS was calculated as the time interval between the start of combined immunotherapy and last contact or death from melanoma. Date of data cut-off analysis was 7th July 2019. RECIST 1.1 criteria were used to assess the response [40]. Progression free survival (PFS) was defined as the time between starting combined immunotherapy and date of disease progression or death. Patients for whom response has not yet been assessed at the time of data cut-off analysis were censored in the PFS analysis and excluded from the analysis of response. For both, MSS and PFS, survival curves were obtained according to the Kaplan–Meier (KM) estimators and compared using the log-rank test, hazard ratios with 95% confidence intervals (95% CIs) were obtained from univariate Cox models. Multiple Cox regression models were constructed using forward variable selection (inclusion *p* = 0.10, exclusion *p* = 0.20) for covariates significant in the univariate analysis. *p*-values from the Cox regression models were obtained from two-sided Wald tests. Odds ratios for response were obtained from univariate logistic regression analyses. The level of significance was 0.05 (two-sided) in each analysis. No correction for multiple testing was applied. Statistical analysis was performed with SPSS v.25 (SPSS Inc.). STATA® v15 (StataCorp LLC, College Station, TX, USA) was used to generate the final version of KM survival curves.

## 3. Results

### 3.1. Patient Characteristics

A total of 59 patients were included, 9 of which harbored a P/LP germline variant. The median age at the time of starting combined immunotherapy was 61 years (IQR 51–74). The majority of the patients had a normal serum lactate dehydrogenase (LDH) (57%), whereas serum S100B was elevated in 60% of the cases; 49% carried a somatic BRAFV600E/K mutation and 78% of the patients had an intermediate-low TMB. Table 2 presents further details on the cohort characteristics.

### 3.2. Pathogenic or Likely Pathogenic Germline Variants Identified

**Figure 1:**
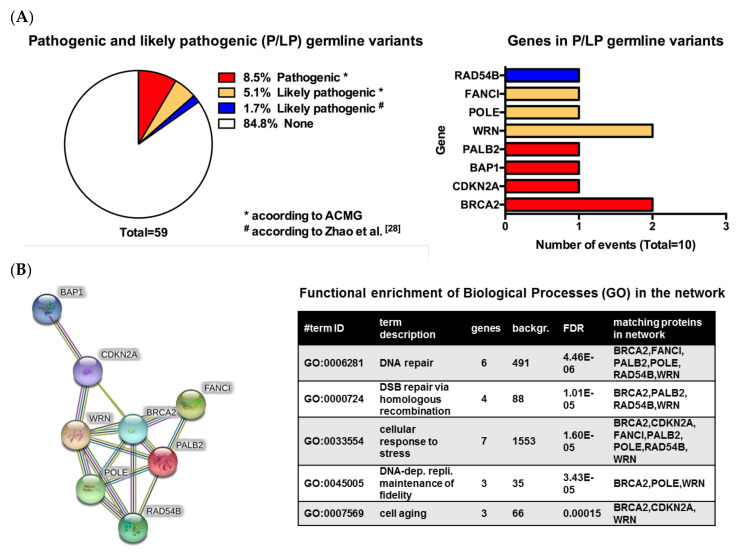

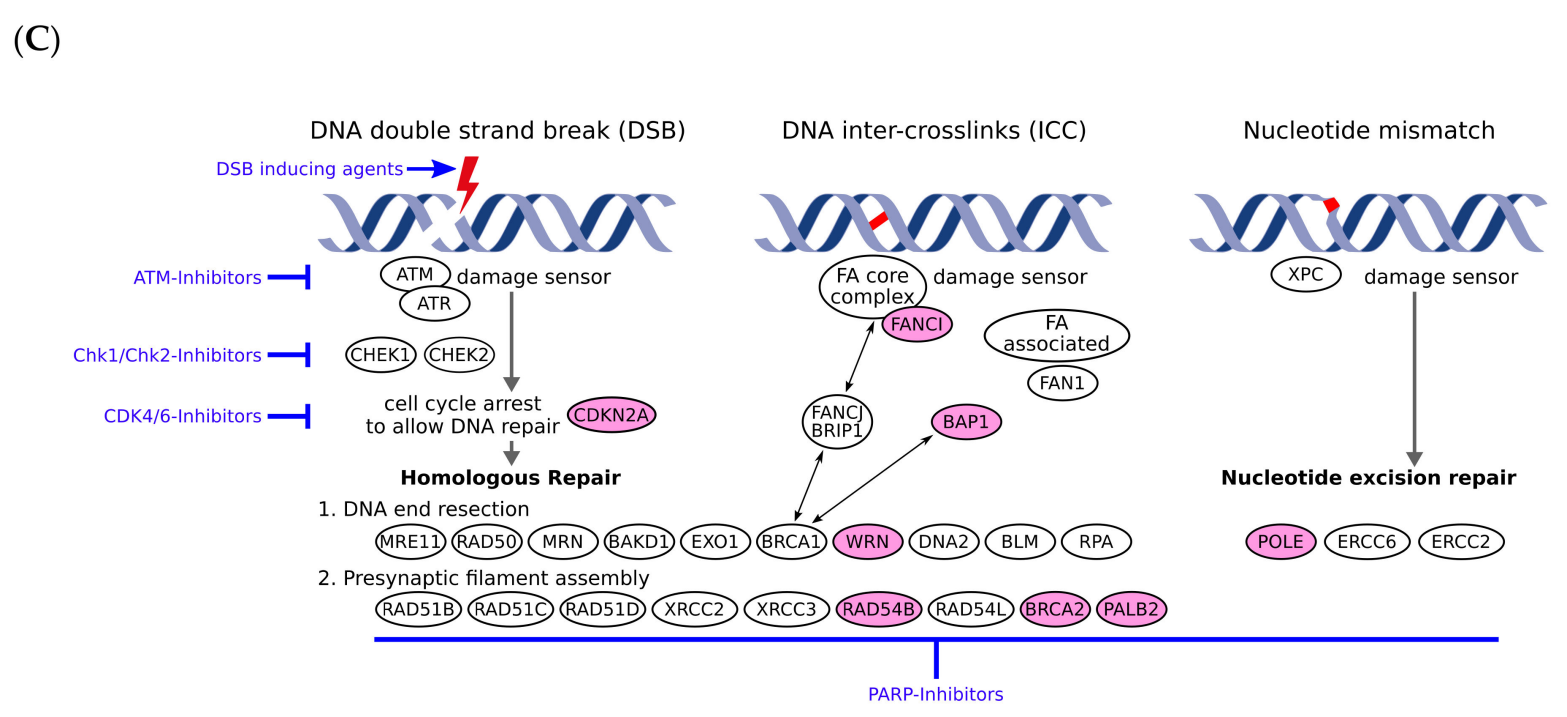
Pathogenic and likely pathogenic germline variants. (**A**) Left pie chart: cohort size by type of detected pathogenic and likely pathogenic (P/LP) germline variants. Each patient is counted according to the most pathogenic variant found (e.g., a patient with a pathogenic variant and a second, likely pathogenic variant is counted in the group of pathogenic variants). Germline variants were found in 15% of patients. Right bar diagram: genes in P/LP germline variants. Most genes harbored mutations in only one patient, while BRCA2 and WRN were affected in two patients each. In one patient two different genes were affected by germline variants both categorized as likely pathogenic (WRN + POLE). Red bars indicate pathogenic variants, yellow indicates likely pathogenic variants and blue shows a non-classifiable variant according to ACMG. (**B**) Left: STRING protein network of the eight different genes with pathogenic and likely pathogenic germline variants showing the known interactions of the corresponding gene products. Right: A table with a functional enrichment analysis of the eight genes with pathogenic and likely pathogenic germline variants using the gene ontology (GO) terms “Biological Process”. The first five top ranked (by the false discovery rate FDR) hits are shown. (**C**) Overview of DNA damage repair pathways with genes found affected in our cohort (highlighted in pink) as well as additional highly relevant genes (selection). Blue labels show targets for targeted therapies. Most pathogenic and likely pathogenic germline variants reported in our cohort are in genes associated with DNA damage repair pathways.

Eight different P/LP germline variants in nine patients were identified, seven of which were involved in DNA repair mechanisms (Table 1). Pathogenic or likely pathogenic germline variants were found in the following genes: *BRCA2*, *CDKN2A*, *BAP1*, *PALB2*, *WRN*, *POLE*, *FANCI* and *RAD54B* (Figure 1A). In one patient, two different likely pathogenic germline variants were found: *POLE* and *WRN* (Table 1). Both patients with a BRCA2 germline variant had additional cancers, one lung cancer, the other one chronic lymphatic leukemia/small cell lymphocytic lymphoma. Furthermore, the BAP1 mutant patient presented with an additional history of clear cell renal cell carcinoma, invasive ductal breast cancer and basal cell carcinoma. The RAD54B mutant patient had additionally prostate cancer. Using the ACMG classification, we identified four different pathogenic variants (*BRCA2*, *CDKN2A*, *BAP1* and *PALB2)* and three likely pathogenic variants (*POLE*, *WRN* and *FANCI*). For reasons described above, we handled the variant in *RAD54B* as a likely pathogenic germline. An in silico analysis using the STRING database revealed the network of the gene products derived of the eight identified genes. Further functional enrichment analysis using the GO biological process terms revealed that indeed the eight genes are majorly involved in DNA repair but also aging and DNA replication and stress response as top enriched processes (Figure 1B). 

### 3.3. Response to Combined Immunotherapy

Thirty-four of the patients treated with combined immunotherapy had progressive disease (PD) and 21 had disease control (DC) that included stable disease (SD), partial response (PR) or complete response (CR), according to RECIST 1.1 criteria (Table 3). Patients included in these two groups (PD and DC) differed significantly in terms of presence of P/LP germline variants (*p* = 0.010) and TMB (*p* < 0.0001), but no significant differences were observed when serum biomarkers S100B and LDH were analyzed (Table 3). All patients with P/LP germline variants that received combined immunotherapy (*n* = 9) had progressive disease.

### 3.4. Survival Analysis

**Figure 2:**
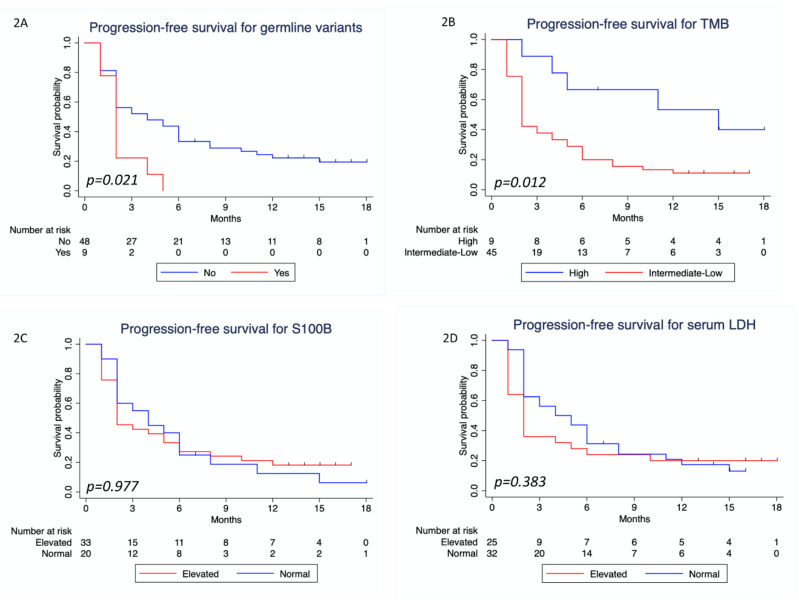
Progression-free survival analysis. (**A**) Kaplan–Meier curve for progression-free survival, considering the presence of pathogenic and likely pathogenic germline variants (Yes/No); (**B**) Kaplan–Meier curve for progression-free survival considering TMB intermediate-low (≤23.1 mut/Mb) or high (>23.1 mut/Mb); (**C**) Kaplan–Meier curve for progression-free survival considering serum S100B levels (normal/elevated); (**D**) Kaplan–Meier curve for progression-free survival considering serum lactate dehydrogenase (LDH) levels (normal/elevated). *p*-values refer to two-sided log rank tests.

The median follow-up time was 13 months (IQR 11–15). Regarding PFS (Table 4), patients with P/LP germline variants did worse compared to those without P/LP germline variants (HR = 2.16; 95% CI: 1.01–4.64; *p* = 0.048). A statistically significant favorable outcome was seen in patients with high TMB (HR = 0.348; 95% CI: 0.14–0.89; *p* = 0.028), but no significant difference was observed when analyzing serum levels of S100B and LDH. Figure 2 shows the Kaplan–Meier PFS curves for these variables. In multivariate Cox regression analysis for PFS, only TMB remained significant (Table 4).

**Figure 3:**
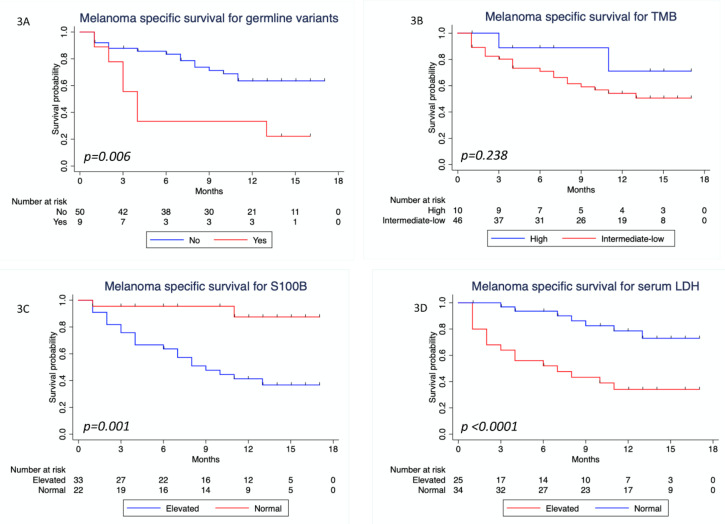
Melanoma specific survival analysis. (**A**) Kaplan–Meier curve for melanoma specific survival, considering the presence of pathogenic and likely pathogenic germline variants (Yes/No); (**B**) Kaplan–Meier curve for melanoma specific survival considering TMB intermediate-low (≤23.1 mut/Mb) or high (>23.1 mut/Mb); (**C**) Kaplan–Meier curve for melanoma specific survival considering serum S100B levels (normal/elevated); (**D**) Kaplan–Meier curve for melanoma specific survival considering serum lactate dehydrogenase (LDH) levels (normal/elevated). *p*-values refer to two-sided log rank tests.

When analyzing MSS, univariate Cox regression analysis showed that there was a statistically significant difference between patients with and without a P/LP germline variant, favoring those without a P/LP germline variant (HR = 3.21; 95% CI: 1.31–7.87; *p* = 0.011; Table 4). A statistically significant difference was also found when analyzing serum S100B and LDH, favoring the group of patients with normal serum levels (*p* = 0.007 and *p* = 0.001, respectively). A trend for better MSS was seen in patients with high TMB (HR = 0.433, 96% CI: 0.10–1.85), but this was not statistically significant (*p* = 0.258). Figure 3 shows the Kaplan–Meier MSS curves for these variables.

In the multivariate Cox regression analysis we found that presence of a P/LP germline variant, serum S100B, and LDH were independently significant for MSS (*p* = 0.036, *p* = 0.044 and *p* = 0.001, respectively; Table 4). 

## 4. Discussion

In our analysis of stage IV melanoma patients treated with nivolumab plus ipilimumab, we found that 15.2% (9/59) of patients harbored a P/LP germline variant, which is in line with what has been reported in other tumor entities so far [9,41] and thus indicates a high prevalence also in melanoma. More importantly, patients with P/LP germline variants presented a significantly worse progression-free survival when compared to the group of patients in which no P/LP germline variant was detected. The same results were found when analyzing melanoma specific survival, suggesting that patients with P/LP germline variants did not benefit from combined immunotherapy. The presence of a P/LP germline variant remained an independently significant factor for melanoma specific survival in the multivariate Cox regression analysis, along with serum S100B and LDH.

When analyzing survival in relation to TMB subgroups (intermediate-low and high), we found that there was a significant difference in PFS favoring patients with high TMB. This is probably explained by the fact that TMB correlates with disease control and response to immunotherapy, as has been shown by our group and others [3,4,31].

In our patient cohort, the majority of P/LP germline variants were present in genes that are associated with pathways involved in DNA repair mechanisms (Figure 1C). We observed that none of the patients with a P/LP germline variant responded to combined immunotherapy, which is currently considered as the most effective therapy in stage IV melanoma [42,43,44]. The explanation why this happened might be related to two mechanisms:

(A) Double strand breaking induced genes and DNA repair genes are involved in the development of immune cells, such as T cells [45,46,47,48,49,50]. On the one hand, it is well known that non-homologous end-joining is decisive for the V(D)J recombination events in developing B- and T-cells where the light and heavy chains of antibodies and T-cell receptors are recombined as random selections of different ready to use DNA segments. Defects in such processes are causal for severe combined immunodeficiency (SCID) syndromes. On the other hand, there are hints that homologous recombination is also involved in lymphocyte development [51]. For example, quiescent hematopoietic stem cells accumulate DNA damage during aging, which needs to be repaired when entering the cell cycle [52]. Defects in the DNA repair mechanisms would accelerate ageing and the accumulation of DNA damage, including the common lymphatic progenitor cells and hence all forms of lymphocytes. This could ultimately lead to a dampened antitumoral immune response and the failure of immune checkpoint inhibitors. Evidence also shows that standard DNA repair mechanisms are involved in immune responses, and defects in these repair mechanisms could translate into deteriorated immune responses and, therefore, worse responses to immunotherapy [45]. The fact that we did not see any response in patients with P/LP germline variants that are involved in DNA repair or homologous recombination adds to this evidence.

(B) It has recently been shown that higher levels of genomic instability were associated with lower immunogenicity in breast cancer patients, which might explain the fact that immune checkpoint inhibitors are less effective in this population [53]. Moreover, it is known that tumors with high genomic instability represented by elevated numbers of copy number variants (CNVs) and a rather low number of single nucleotide variants (SNVs) respond worse to immune checkpoint blockade [54]. There might be a similar mechanism present in the advanced melanoma patients included in our study, which could explain the absence of response and worse survival of these patients. 

In this context, one needs to consider whether some patients with P/LP germline variant might be better served with systemic therapies that include targeted therapies, namely PARPi, and/or cytotoxic chemotherapies and not immunotherapy alone [55]. Platinum compounds that act by targeting the DNA repair mechanisms [56] might be considered a potential combination therapy in future trials including this subgroup of patients, similar to what has been previously shown and proposed in other tumor entities [55,57].

Presently, there is methodological diversity for sequencing DNA from tumor and normal tissue, namely whole genome, whole exome and panel sequencing with the latter showing the highest diagnostic sensitivity with respect to germline and subclonal variants [58,59]. In our study, both tumor and normal DNA have been sequenced deeply, focusing on >700 tumor relevant genes, in order to identify both germline and somatic variants.

Besides the controversy regarding the best sequencing method, there is also no consensus approach for reporting the results, particularly for germline variants. Reporting germline variants is an important aspect when considering the potential implications not only for the therapy of the patients, but also for their relatives. The ACMG classification is, as their authors mentioned, a work in progress [26]. Our understanding is that even if some variants cannot be classified as pathogenic, they should not be excluded from the genetic report, particularly when associated with a potentially increased risk or indicating that a targeted therapy can be offered. This was the concept in the current report.

Our study has a clear limitation regarding the small number of cases included. Therefore, the analyses have a limited statistical power, and should be interpreted with caution. A trend towards a worse prognosis for patients with P/LP germline variants can be seen from our data; none of the patients with a P/LP germline variant responded to combined immunotherapy, but the low number (*n* = 9) does not allow us to draw further conclusions. 

## 5. Conclusions

The main finding of our study is that the presence of P/LP germline variants appeared to be correlated with primary resistance to combined immunotherapy, shorter progression-free survival and worse melanoma specific survival in melanoma patients treated with combined immunotherapy. The majority of the P/LP germline variants identified concerns genes associated with DNA repair mechanisms. Further, these mutated genes are frequently involved in processes needed for lymphocyte development and an impairment of such processes could lead to a diminished immune response and resistance to immunotherapy by immune checkpoint inhibitors. Our hypothesis needs to be further evaluated in a larger multicentric cohort and ideally in a future clinical trial.

## Figures and Tables

**Table 1:** Pathogenic and likely pathogenic germline variants reported.

Patient No.	Melanoma Subtype	Gene Name	Germline Variant	ACMG Classifiable [1]	Rationale	TMB (mut/Mb)	Pathway Involved	Potential Therapy	LOE^$^
1	Cutaneous (nodular)	BRCA2	c.6446_6449delTTAA; p.Ile2149Lysfs*18 (het.), NM_000059.3	Class 5	Pathogenic germline variant	40.9	DNA repair, homologous recombination	PARPi	2A
2	Cutaneous (nodular)	POLE	c.1270C>T; p.Leu424Phe (het.), NM_006231.3	Class 4	Likely pathogenic germline variant	21.4	DNA repair, homologous recombination	PARPi	2B
								Immunotherapy	1B
		WRN	c.2893_2899del; p.Lys965Cysfs*7 (het.), NM_000553.4	Class 4	Likely pathogenic germline variant		Homologous recombination	PARPi	2B
3	Acral	FANCI	CNV coding exon 9 (het. Deletion), NM_001113378.1	Class 4	Likely pathogenic germline variant	1.6	DNA repair, homologous recombination	PARPi	4
4	Cutaneous (superficial)	WRN	c.348delC; p.Met117Cysfs*8 (het.), NM_000553.4	Class 4	Likely pathogenic germline variant	7.9	Homologous recombination	PARPi	2B
5	Cutaneous (polypoid)	CDKN2A	c.301G>T; p.Gly101Trp (het.), NM_000077.4	Class 5	Pathogenic germline variant	5.8	Cell cycle	CDK 4/6 inhibitors	2B
6	Uveal	BAP1	c.37+1G>T; p.? (het.), NM_004656.3	Class 5	Pathogenic germline variant	1.0	Homologous recombination, epigenetics	PARPi	2B
								EZH2i	3
								HDACi	4
7	Cutaneous (superficial)	PALB2	c.757_758delCT; p.Leu253Ilefs*3 (het.), NM_024675.3	Class 5	Pathogenic germline variant	3.1	Homologous recombination	PARPi	2B
8	Cutaneous (nodular)	BRCA2	c.1888dupA; p.Thr630Asnfs*6 (het.), NM_000059.3	Class 5	Pathogenic germline variant	12.0	DNA repair, homologous recombination	PARPi	2A
9	Cutaneous (nodular)	RAD54B	c.889C>T; p.Arg297* (het.), NM_012415.3	No	Likely pathogenic germline variant/ Potentially therapy relevant	13.1	DNA repair, homologous recombination	PARPi	4

ACMG = American College of Medical Genetics and Genomics; LOE= level of evidence—please refer to Supplementary Materials supplement 2; PARPi = adenosine diphosphate (ADP) ribose polymerase (PARP) inhibitors; EZH2i = enhancer of zeste homolog 2 inhibitors; HDACi = histone deacetylase inhibitors; CDK = cyclin-dependent kinases. **^$^** Please refer to Supplementary Materials supplement 2.

**Table 2:** Patients’ characteristics.

Characteristic	*N* (%)
**Sex**	
Male	36 (61)
Female	23 (39)
**Age**	
Primary disease diagnosis (median years; IQR)	58; (49–70)
Initiation of combined immunotherapy (median years; IQR)	61; (51–74)
**Melanoma subtype**	
Cutaneous melanoma	35 (59.3)
Acral melanoma	6 (10.2)
Mucosal melanoma	4 (6.7)
Melanoma of unknown primary	5 (8.5)
Uveal melanoma	9 (15.3)
Tumor mutation burden* (*n* = 56)	
TMB (mut/Mb median; IQR)	4.7; (1.7–13.97)
Intermediate-low (≤23.1 mut/Mb)	46 (82.1)
High (>23.1 mut/Mb)	10 (17.9)
**Serum biomarkers**	
LDH	
Normal	34 (57.6)
Elevated	25 (42.4)
S100B* (*n* = 55)	
Normal	22 (40)
Elevated	33 (60)
BRAF mutation* (*n* = 57)	
Yes	29 (50.9)
No	28 (49.1)
**Metastasis at start of nivolumab plus ipilimumab**	
Presence of cerebral metastasis	24 (40.7)
Presence of liver metastasis	17 (28.8)
Presence of lung metastasis	32 (54.2)
**Nivolumab plus ipilimumab**	
First line	29 (49.2)
Second line or more	30 (50.8)
Response to nivolumab and ipilimumab at first staging* (*n* = 55)	
Progressive disease	34 (61.8)
Disease control	21 (38.2)

* Denotes variables for which patients with missing values were excluded.

**Table 3:** Response to combined immunotherapy dependent on potential predictors (*n* = 55).

Category	Disease Control *N* = 21	Progressive Disease *N* = 34	*p*	Odds Ratio (95% CI)
	*N* (%)			
**Pathogenic and likely pathogenic germline variants**				
Present	0	9 (26.5)	0.010	NA*
Not present	21 (100)	25 (73.5)		
**Tumor mutation burden** **(*n* = 52)**				
Low /Intermediate	11 (57.9)	32 (97)	<0.0001	23.27 (2.61–207.7)
High	8 (42.1)	1 (3)		
**Serum biomarkers**				
**S100B (*n* = 51)**				
Normal	9 (47.4)	11 (34.4)	0.36	
Elevated	10 (52.6)	21 (65.6)		1.72 (0.54–5.47)
**LDH**				
Normal	15 (71.4)	17 (50)	0.118	
Elevated	6 (28.6)	17 (50)		2.50 (0.78–7.98)

Two-sided exact Pearson Chi-square test (*p*-value) and logistic regression (odds ratio). * Not available due to zero patients with pathogenic or likely pathogenic germline variants in the disease control group, risk difference: 100% − 54% = 46%.

**Table 4:** Progression-free survival and melanoma specific survival dependent on potential predictors.

Category	Progression-Free Survival				Melanoma Specific Survival				
	HR (95% CI)*^1^*	*p* ^1^	HR (95% CI)^2^	*p* ^2^	HR (95% CI)^1^	*p* ^1^	HR (95% CI)^2^	*p* ^2^	
**Pathogenic and likely pathogenic germline variants**									
Present vs. not present	2.16 (1.01–4.64)	0.048	1.93 (0.89–4.15)	0.095	3.21 (1.31–7.87)	0.011	2.93 (1.07–8.0)		0.036
**Tumor mutation burden**									
Low/intermediate vs. high	2.88 (1.12–7.38)	0.028	2.75 (1.07–7.09)	0.036	2.31 (0.54–9.85)	0.258	NA		
**S100B**									
Elevated vs. normal	0.992 (0.55–1.81)	0.979	NA		7.45 (1.74–31.91)	0.007	4.65 (1.04–20.76)		0.044
**LDH**									
Elevated vs. normal	1.26 (0.71–2.26)	0.433	NA		4.33 (1.77–10.56)	0.001	5.16 (1.90–13.96)		0.001

^1^ Univariate Cox regression analysis; ^2^ multivariate Cox regression analysis for variables significant in the univariate analysis. NA variable was not included in the multivariate Cox regression analysis. *p*-values refer to the two-sided Wald test.

## Supplementary Materials

The following are available online at https://www.mdpi.com/2072-6694/12/5/1101/s1, Supplement 1: The 710 genes of the tumor panel, Supplement 2: Level of evidence (LOE) existing for each treatment option and used to classify information in table 1.
