# Supplementary material for: Are Pathogenic Germline Variants in Metastatic Melanoma Associated with Resistance to Combined Immunotherapy?
Source: Cancers (Basel). 2020 Apr 28;12(5):1101. doi: 10.3390/cancers12051101 (PMC7281129; doi:10.3390/cancers12051101)
Supplement: Supplementary file 1 [file cancers-12-01101-s001.pdf]

Supplementary material

Supplement 1: The 710 genes of the tumor panel

ABL1, ABL2, ACD, AIP, AJUBA, AKT1, AKT2, AKT3, ALK, AMER1, ANKRD26, APC, AR, ARAF, ARHGAP35, ARID1A, ARID1B, ARID2, ARID5B, ASXL1, ASXL2, ATG2B, ATM, ATP1A1, ATR, ATRX, AURKA, AURKB, AURKC, AXIN1, AXIN2, AXL, B2M, BAP1, BARD1, BCL10, BCL11A, BCL11B, BCL2, BCL3, BCL6, BCL9, BCOR, BCORL1, BCR, BIRC2, BIRC3, BIRC5, BLM, BMPR1A, BRAF, BRCA1, BRCA2, BRD3, BRD4, BRIP1, BTK, BTNL2, BUB1B, C11ORF30, CALR, CAMK2G, CARD11, CASP8, CBFB, CBL, CBLB, CBLC, CCDC6, CCND1, CCND2, CCND3, CCNE1, CD274, CD38, CD52, CD58, CD79A, CD79B, CD82, CDC73, CDH1, CDH11, CDH2, CDK12, CDK4, CDK6, CDK8, CDKN1A, CDKN1B, CDKN1C, CDKN2A, CDKN2B, CDKN2C, CEBPA, CEP57, CHD1, CHD2, CHD4, CHEK1, CHEK2, CIC, CIITA, CKS1B, CNOT3, COL1A1, COMMD1, CREB1, CREBBP, CRKL, CRTC1, CRTC2, CSF1R, CSF2, CSF3R, CSMD1, CSNK1A1, CTCF, CTLA4, CTNNA1, CTNNB1, CUL4B, CUX1, CXCR4, CYLD, CYP2A7, DAXX, DCC, DDB2, DDR1, DDR2, DDX11, DDX3X, DDX41, DEK, DHFR, DICER1, DIS3, DIS3L2, DKC1, DNMT1, DNMT3A, DOT1L, DPYD, EBP, EGFR, EGLN1, EGR2, EGR3, ELAC2, ELANE, ELF3, EML4, EP300, EPAS1, EPCAM, EPHA2, EPHA3, EPHA4, EPHB4, EPHB6, ERBB2, ERBB3, ERBB4, ERCC1, ERCC2, ERCC3, ERCC4, ERCC5, ERG, ERRFI1, ESR1, ESR2, ETNK1, ETS1, ETV1, ETV4, ETV5, ETV6, EWSR1, EXO1, EXT1, EXT2, EZH1, EZH2, FAM175A, FAM46C, FAN1, FANCA, FANCB, FANCC, FANCD2, FANCE, FANCF, FANCG, FANCI, FANCL, FANCM, FAS, FAT1, FBXW7, FES, FGF10, FGF14, FGF19, FGF2, FGF23, FGF3, FGF4, FGF5, FGF6, FGFBP1, FGFR1, FGFR2, FGFR3, FGFR4, FH, FKBP1A, FLCN, FLI1, FLT1, FLT4, FOXA1, FOXA2, FOXE1, FOXL2, FOXO1, FOXO3, FOXP1, FOXQ1, FRK, FRS2, FUBP1, FUS, FYN, G6PD, GABRA6, GALNT12, GATA1, GATA2, GATA3, GATA4, GATA6, GLDN, GLI1, GLI2, GNA11, GNA13, GNAQ, GNAS, GPC3, GPER1, GPR124, GREM1, GRIN2A, GRM3, GSK3A, H3F3A, HCK, HGF, HIF1A, HIST1H3B, HLA-A, HLA-B, HLA-C, HLA-DPA1, HLA-DPB1, HLA-DQA1, HLA-DQB1, HLA-DRA, HLA-DRB1, HLF, HMGA2, HMGN1, HMOX2, HNF1A, HNF1B, HOXB13, HOXD8, HRAS, HSD3B1, HSP90AA1, HSP90AB1, ID3, IDH1, IDH2, IFNGR1, IFNGR2, IGF1R, IGF2, IGF2R, IKBKB, IKBKE, IKZF1, IKZF3, IL1B, IL1RN, IL2, IL21R, IL6, IL6ST, IL7R, ING4, INPP4B, INPPL1, IRF1, IRS2, ITK, JAK1, JAK2, JAK3, JUN, KAT6A, KDM5A, KDM5C, KDM6A, KDR, KEAP1, KIAA1549, KIT, KLF2, KLF4, KLHDC8B, KLHL6, KMT2A, KMT2B, KMT2C, KMT2D, KRAS, LATS1, LATS2, LCK, LIG4, LIMK2, LMO1, LRP1B, LRRK2, LTK, LYN, LZTR1, MAD2L2, MAFB, MAGEA1, MAGI1, MAGI2, MAML1, MAP2K1, MAP2K2, MAP2K3, MAP2K4, MAP2K5, MAP2K6, MAP2K7, MAP3K1, MAP3K14, MAP3K3, MAP3K4, MAP3K6, MAPK1, MAPK11, MAPK12, MAPK3, MAPK8IP1, MAX, MBD1, MC1R, MCL1, MDC1, MDM2, MDM4, MECOM, MED12, MEF2B, MEN1, MET, MGA, MGMT, MITF, MLH1, MLH3, MLLT10, MLLT3, MN1, MPL, MRE11A, MS4A1, MSH2, MSH3, MSH4, MSH5, MSH6, MSR1, MST1R, MTHFR, MTOR, MTRR, MUC1, MUC16, MUTYH, MXI1, MYB, MYC, MYCL, MYCN, MYD88, MYH11, MYH9, NBN, NCOA1, NCOA3, NCOR1, NF1, NF2, NFE2L2, NFKB1, NFKB2, NFKBIA, NFKBIE, NIN, NLRC5, NOP10, NOTCH1, NOTCH2, NOTCH3, NOTCH4, NPM1, NQO1, NR1I3, NRAS, NRG2, NSD1, NT5C2, NTHL1, NTRK1, NTRK2, NTRK3, NUMA1, NUP98, PAK3, PALB2, PALLD, PARK2, PARP1, PARP2, PARP4, PAX3, PAX5, PAX7, PBK, PBRM1, PBX1, PDCD1, PDCD1LG2, PDF, PDGFA, PDGFB, PDGFC, PDGFD, PDGFRA, PDGFRB, PDK1, PGR, PHF6, PHOX2B, PIAS4, PIGA, PIK3C2A, PIK3C2B, PIK3C2G, PIK3CA, PIK3CB, PIK3CD, PIK3CG, PIK3R1, PIK3R2, PIK3R3, PIM1, PKHD1, PLCG1, PLCG2, PML, PMS1, PMS2, POLD1, POLE, POLH, POLQ, POT1, PPM1D, PRDM1, PRDM16, PREX2, PRF1, PRKAR1A, PRKCA, PRKD1, PRKDC, PROM2, PRSS1, PRX, PSIP1, PSMB1, PSMB10, PSMB2, PSMB5, PSMB8, PSMB9, PSMC3IP, PSPH, PTCH1, PTCH2, PTEN, PTGS2, PTK2, PTK7, PTPN11, PTPRC, PTPRD, PTPRT, RAC1, RAC2, RAD21, RAD50, RAD51, RAD51B, RAD51C, RAD51D, RAD54B, RAD54L, RAF1, RALGDS, RARA, RARB, RARG, RASA1, RASAL1, RB1, RBM10, RECQL4, REL, RET, RFC2, RFX5, RHBDF2, RHEB, RHOA, RICTOR, RINT1, RIPK1, RIT1, RNASEL, RNF2, RNF43, ROS1, RPL22, RPS20, RPS6KB1, RPTOR, RSF1, RUNX1, RYR1, SACS, SAMHD1, SAV1, SBDS, SCG5, SDHA, SDHAF2, SDHB, SDHC, SDHD, SEC23B, SEMA4A, SETBP1, SETD2, SETDB1, SF3B1, SGK1, SH2B1, SH2B3, SH2D1A, SHFM1, SHH, SIK2, SIN3A, SIRT1, SKP2, SLC26A3, SLIT2, SLX4, SMAD3, SMAD4, SMARCA4, SMARCB1, SMARCE1, SMC1A, SMC3, SMO, SOCS1, SOX11, SOX2, SOX9, SPEN, SPINK1, SPOP, SPRED1, SPTA1, SRC, SRD5A2, SRGAP1, SRP72, SRSF2, SSTR1, SSTR2, SSTR3, SSTR5, SSX1, STAG1, STAG2, STAT1, STAT3, STAT5A, STAT5B, STK11, SUFU, SUZ12, SYK, TAF1, TAF15, TAP1, TAP2, TBK1, TBL1XR1, TBX3, TCF3, TCF7L2, TCL1A, TEK, TERC, TERF2IP, TERT, TET1, TET2, TFE3, TGFBR2, TLR4, TLX1, TMEM127, TNF, TNFAIP3, TNFRSF11A, TNFRSF13B, TNFRSF14, TNFRSF1A, TNFRSF1B, TNFRSF25, TNFRSF8, TNFSF11, TNK2, TOP1, TOP2A, TP53, TP53BP1, TPX2, TRAF2, TRAF3, TRAF5, TRAF6, TRAF7, TRRAP, TSC1, TSC2, TSHR, TUBA4A, TUBB, TYMS, U2AF1, UBE2T, UBR5, UGT2B15, UGT2B7, UIMC1, UNG, USP34, USP9X, VEGFA, VEGFB, VHL, VKORC1, WAS, WASF3, WHSC1, WISP3, WRN, WT1, XIAP, XPA, XPC, XPO1, XRCC1, XRCC2, XRCC3, XRCC5, XRCC6, YAP1, ZFHX3, ZHX3, ZNF217, ZNRF3, ZRSR2

Supplement 2: Level of evidence (LOE) existing for each treatment option and used to classify information in table 1

LOE	
**1A**	**Approved drug, specific to the biomarker and entity**<br>Drug is approved for the biomarker within the same entity (FDA and/or EMA)
**1B**	**Approved drug, specific to entity but not specific to the biomarker OR specific to biomarker, but only in organ related entities**<br>Drug is approved independently of the biomarker within the same entity OR drug is approved for the biomarker in an organ related entity, e. g. benign tumor (FDA and/or EMA). The reported biomarker must have significant clinical relevance, despite biomarker-independent approval of the indicated drug.
**2A**	**Approved drug, specific to the biomarker for a different entity**<br>Drug is approved for the biomarker in a different entity (FDA and/or EMA)
**2B**	**Approved drug, not specific to the biomarker for a different entity**<br>Drug is approved independently of the biomarker in a different entity (FDA and/or EMA). The reported biomarker must have significant clinical relevance, despite biomarker-independent approval of the indicated drug.
**3**	**Efficacy of the drug is currently being/was analysed in clinical trials**
**4**	**Efficacy of the drug is based on preclinical analyses and/or case reports**
**5**	**Hypothetical response**<br>The biomarker could hypothetically induce response to the drug
**R1**	**The variant and/or biomarker is associated with a non-response, decreased response, or resistance to a specific drug or drug class in the same entity. The information is based on high impact guidelines (NCCN and/or ESMO)**<br>The variant and/or biomarker is associated with a non-response, decreased response, or resistance to a specific drug or drug class in the same entity. The information is based on high impact guidelines (NCCN and/or ESMO)
**R2**	**Biomarker might be associated with a non-response or a resistance**<br>The biomarker might be associated with a non-response, reduced response, or resistance to the stated drug class in this or another tumor entity (based on current literature)

Please note, the LOE classification reported here is adapted from the LOE classification established by the oncology knowledge base (OncoKB) [38].
